# Supplementary material for: Lotus (Nelumbo nucifera Gaertn.) Leaf-Fermentation Supernatant Inhibits Adipogenesis in 3T3-L1 Preadipocytes and Suppresses Obesity in High-Fat Diet-Induced Obese Rats
Source: Nutrients. 2022 Oct 17;14(20):4348. doi: 10.3390/nu14204348 (PMC9610561; doi:10.3390/nu14204348)
Supplement: Supplementary file 1 [file nutrients-14-04348-s001.zip › nutrients-1911079-supplementary.pdf]

Table S1. The sequence of primers used for RT-qPCR.

Primer name		Forward primer (5’-3’)	Reverse primer (5’-3’)
Cell	*C/EBPα*	CAAGAACAGCAACGAGTACCG	AGGCGGTCATTGTCACTGGT
	C/EBPβ	AGAAGACGGTGGACAATGTGA	GTCAGCTCCAGCACCTTGTG
	FAS	TGGTGGTGTGGACATGGTCACAGA	CCGAAGCTGGGGGTCCATTGTGTG
	ACC	CCCAGCAGAATAAAGCTACTTTGG	TCCTTTTGTGCAACTAGGAACGT
	GAPDH	ACAACTTTGGTATCGTGGAAGG	GCCATCACGCCACAGTTTC
Rat	*C/EBPα*	GGAGACGCAGCAGAAGGTGTTG	GTGTCCAGTTCACGGCTCAGC
	PPARγ	TGTGGGGATAAAGCATCAGG	CAAGGCACTTCTGAAACCGA
	ACC	TTGTGGATGGCTTGCGGGAATG	GTGCCGAGGATTGATGGTTGGG
	FAS	TTTCATCAGGCCACCATCCT	ATCAGGTTGCCACTGTCAGA
	β-actin	CACCATGTACCCAGGCATTG	CACCATGTACCCAGGCATTG
